# Carboxymethyl cellulose from oil palm trunk slabs: physicochemical properties and performance in acyclovir buccal films

**DOI:** 10.1039/d6ra04937k

**Published:** 2026-11-10

**Authors:** Chutima Jantarat, Sirikanya Kaewpradit, Jiraporn Chingunpitak, Suthon Srivaro

**Affiliations:** a Drug and Cosmetics Excellence Center, Walailak University Thasala Nakhon Si Thammarat 80160 Thailand chutima.ja@wu.ac.th; b School of Pharmacy, Walailak University Thasala Nakhon Si Thammarat 80160 Thailand; c Center of Excellence in Wood and Biomaterials, Walailak University Thasala Nakhon Si Thammarat 80160 Thailand

## Abstract

Carboxymethyl cellulose (CMC) is widely used as a pharmaceutical excipient in mucoadhesive and film-forming drug delivery systems. This study aimed to synthesize CMC from oil palm trunk slab (OPTS) waste obtained from the bottom and middle sections of oil palm trunks and evaluate its potential for use in acyclovir-loaded buccal films. Cellulose was extracted from OPTS by dewaxing, alkali treatment, and hydrogen peroxide bleaching, followed by carboxymethylation with sodium monochloroacetate. The resulting CMC samples were characterized by Fourier transform infrared spectroscopy, differential scanning calorimetry, scanning electron microscopy, degree of substitution, viscosity, moisture content, and pH. Acyclovir-loaded buccal films were prepared using the synthesized CMC and evaluated for physicochemical properties, mucoadhesion, drug release, and *ex vivo* buccal permeation in comparison with films containing commercial CMC. The cellulose yields from the bottom and middle sections of OPTS were 41.43 ± 0.25% and 28.15 ± 0.33%, respectively. The synthesized CMC samples showed successful carboxymethylation, moderate degrees of substitution, and lower viscosity than commercial CMC. Buccal films prepared with the synthesized CMC exhibited satisfactory physicochemical properties and comparable mucoadhesive force, drug release, and *ex vivo* permeation behavior to films containing commercial CMC, although their mucoadhesive residence times were shorter. These findings indicate that OPTS waste can serve as a potential source of CMC for buccal film formulations, while further optimization of polymer viscosity and mucoadhesive retention is needed for applications requiring prolonged buccal residence. The higher cellulose yield obtained from the bottom section also indicates its potential as a productive raw material for CMC production.

## Abstract 2

Oil palm trunk slab waste was converted into carboxymethyl cellulose for acyclovir-loaded buccal films, demonstrating its potential as a sustainable pharmaceutical excipient.

## Graphical abstract

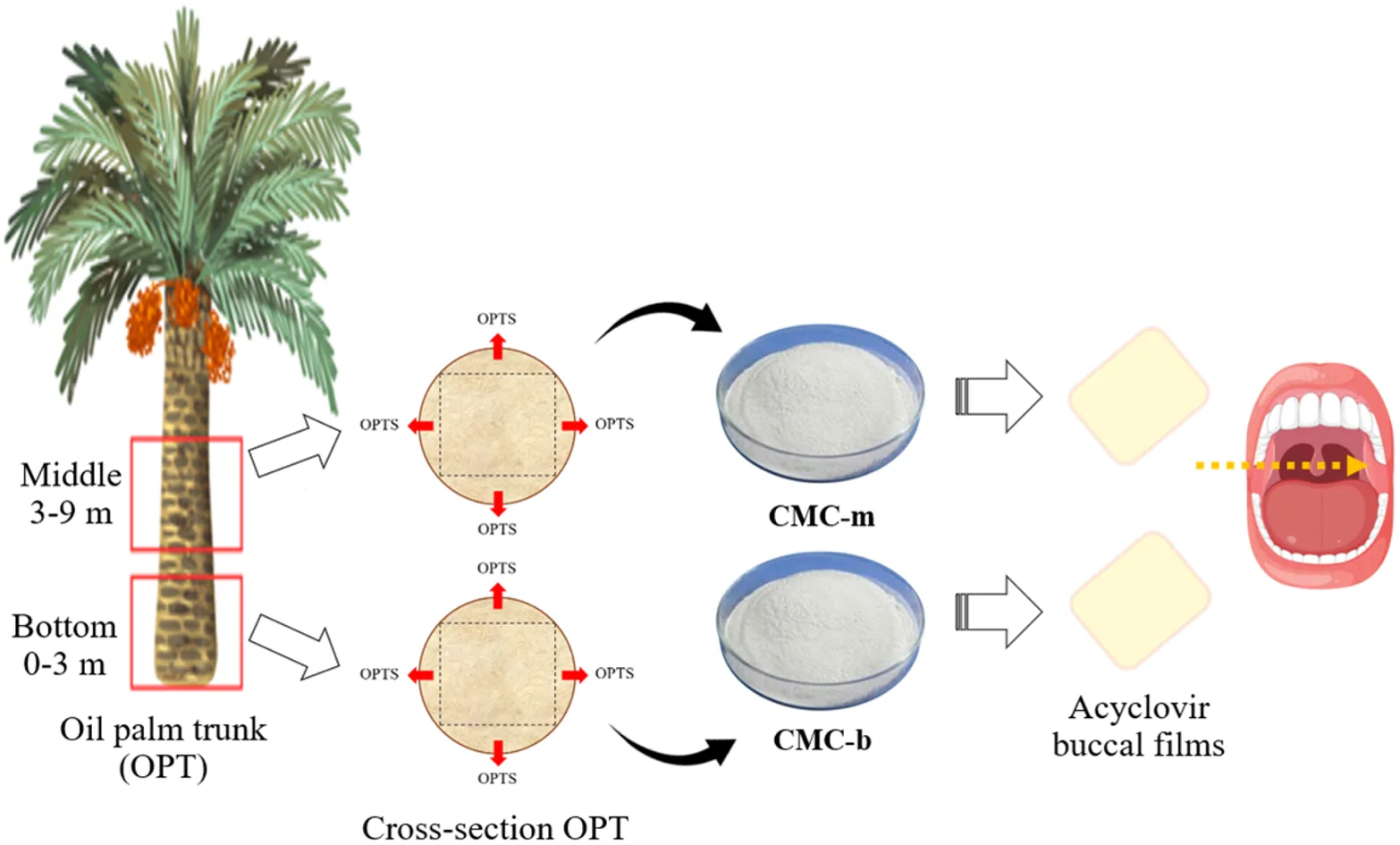

## Introduction

Carboxymethyl cellulose (CMC) is a semi-synthetic derivative of cellulose, a naturally abundant biopolymer primarily found in plant cell walls. It is produced *via* chemical modification in which hydroxyl groups (–OH) on the cellulose backbone are partially substituted with carboxymethyl groups (–CH_2_–COOH), resulting in a water-soluble polymer with enhanced functional properties.^1^ CMC typically appears as a white to off-white powder and readily dissolves in both cold and hot water to form viscous solutions. Owing to its favourable physicochemical characteristics, including biocompatibility, non-toxicity, and functional versatility, CMC is widely utilized in pharmaceutical formulations as an emulsifying agent, gel-forming agent, and tablet binder, as well as in injectable and oral solution dosage forms.^2,3^ In particular, CMC has gained considerable attention as a key excipient in buccal drug delivery systems due to its excellent mucoadhesive, film-forming, and hydrophilic properties, which facilitate prolonged residence time in the oral cavity and enhance drug absorption across the buccal mucosa.^4^ However, the performance of CMC in pharmaceutical applications is strongly influenced by parameters such as molecular weight, degree of substitution (DS), and viscosity, which are, in turn, dependent on the cellulose source and synthesis conditions, including alkalization and etherification processes.^5–7^

In recent years, the synthesis of CMC has increasingly focused on sustainable and renewable resources, particularly agricultural waste, in response to environmental concerns and the growing emphasis on green chemistry.^8^ Traditionally, CMC is derived from purified cellulose sources such as wood pulp or cotton linters; however, these resources are associated with sustainability concerns, prompting a shift toward alternative lignocellulosic feedstocks.^9–11^ Among these, oil palm biomass has emerged as a promising candidate due to its abundance and relatively high cellulose content. Oil palm residues, including empty fruit bunches (EFB), fronds, and oil palm trunks (OPT), collectively generate substantial quantities of biomass annually, exceeding 200 million tons worldwide.^12^ The cellulose content of these materials typically ranges from 30% to 50%, making them suitable for cellulose-based applications.^13^ EFB, in particular, has been extensively studied as a raw material for cellulose and CMC production.^14–17^ In contrast, OPT biomass, which is generated during replanting cycles when oil palm trees reach approximately 25 years of age, remains relatively underexplored for CMC synthesis. Current disposal practices for OPT, such as open burning or chemical treatment, may lead to environmental and health concerns.^18^ Unlike EFB, which is generated during fruit processing, OPT is obtained from the trunk during plantation replanting and, therefore, represents a distinct and potentially complementary source of oil palm-derived cellulose. Importantly, processing of OPT generates trunk slabs (OPTS), particularly from the outer portion of the trunk, which are often underutilized despite their relatively high cellulose content.^19,20^ The use of OPTS could therefore provide an opportunity to valorize an underutilized trunk-derived biomass stream while diversifying the raw material sources available for cellulose and CMC production. This is particularly relevant for pharmaceutical applications, where the properties of CMC depend strongly on the characteristics of its cellulose precursor.^21^

Although the chemical structure of CMC is fundamentally similar, variations in the properties of the raw cellulose such as source purity, crystallinity, and DS can significantly influence the physical and functional characteristics of the final product.^22^ Consequently, CMC derived from different sources exhibits distinct physicochemical properties.^11^ This variability is also observed within oil palm biomass. For example, CMC derived from EFB typically exhibits DS values in the range of 0.2–0.7, whereas CMC derived from OPT has been reported to reach DS values as high as 1.45 under comparable synthesis conditions.^19,23,24^ Since DS directly affects viscosity and functional performance, such variations are critical for pharmaceutical applications. To date, CMC derived specifically from OPTS has not been reported, and its physicochemical properties remain largely unexplored. Given that OPTS possesses a softer structure compared to conventional lignocellulosic sources such as wood, cellulose extraction is expected to be more efficient, further supporting its potential as a sustainable raw material for CMC production. Although EFB has been widely investigated as an oil palm-derived cellulose source, using OPTS provides an alternative approach to valorizing trunk biomass generated during plantation replanting. In addition, the relatively soft, cellulose-rich nature of the OPTS fractions investigated in this study may facilitate processing and conversion into cellulose derivatives.^19,20^ However, whether OPTS can provide CMC with physicochemical properties suitable for pharmaceutical applications remains unclear and requires systematic evaluation.

Acyclovir is a widely used antiviral agent for the treatment of infections caused by herpes viruses, including herpes simplex and varicella-zoster viruses.^25^ Despite its therapeutic efficacy, acyclovir exhibits low oral bioavailability (approximately 10–20%) and a short elimination half-life (approximately 2.5–3.3 h), necessitating frequent dosing (up to five times daily), which can reduce patient compliance.^26,27^ Various drug delivery strategies have therefore been explored to overcome these limitations, including controlled-release systems, transdermal devices, and mucoadhesive formulations.^28–31^ Among these, buccal drug delivery systems offer a promising approach by enhancing drug residence time and absorption while bypassing first-pass metabolism. Suitable drug candidates for buccal delivery typically possess moderate molecular weight, balanced lipophilicity, and adequate stability; acyclovir meets several of these criteria, supporting its suitability for this route of administration.^32^

Therefore, this study investigates the synthesis and characterization of CMC derived from OPTS obtained from the bottom and middle sections of the trunk, and evaluates its potential application for the development of acyclovir buccal drug delivery systems. The physicochemical properties of CMC synthesized from OPTS using conventional methods were first characterized with emphasis on parameters relevant to pharmaceutical applications, particularly buccal film formulation. In addition, CMC obtained from different OPTS regions was comparatively evaluated alongside commercial CMC (CMC®). Finally, the synthesized CMC was assessed for its suitability as a mucoadhesive and film-forming agent in acyclovir-loaded buccal films to determine its potential as a sustainable pharmaceutical excipient.

## Materials and methods

### Materials

Oil palm trunk slabs (OPTS) were obtained from oil palm trunk (OPT) approximately 25 years old, collected from an oil palm plantation in Thasala District, Nakhon Si Thammarat Province, Thailand. The trunks were sampled from the bottom (0–3 m) and middle (3–6 m) sections measured from ground level. Each section was cut crosswise into slices approximately 5 cm thick. The bark was removed, and the trunks were cut into square sections to simulate conventional wood lumber processing. OPTS, representing the outer portion generated during processing, were collected and further cut into pieces approximately 2 cm wide and 5 cm long. The OPTS pieces were rinsed with distilled water and dried in a hot-air oven at 55 °C for 72 h. The dried material was ground into powder using a grinder (PG-ECO-0300, Spring Green Evolution Co., Ltd, Thailand). Vascular bundles were mechanically removed, and the resulting powder was sieved through a 40-mesh sieve to obtain the parenchyma fraction. The powders derived from the bottom and middle sections were designated as OPTS-b and OPTS-m, respectively, and stored in a desiccator until further use.

Specific chemicals used in this study included sodium monochloroacetate (SMCA), purchased from Loba Chemie (Mumbai, India); carboxymethylcellulose, used as a reference compound and hereafter referred to as CMC®, purchased from Merck (Darmstadt, Germany); and acyclovir, obtained from Chanjao Longevity Co., Ltd (Bangkok, Thailand). All other chemicals and reagents were of analytical grade and used as received.

### Extraction of cellulose from OPTS and synthesis of CMC

Cellulose was extracted from OPTS prior to its conversion into carboxymethylcellulose (CMC). The extraction process consisted of three sequential steps: dewaxing, hemicellulose removal, and delignification, following a previously reported method.^20^ To remove residual wax, 80 g of OPTS powder was treated with 450 mL of an ethanol/toluene solution (2 : 1, v/v) under continuous stirring at 250 rpm at 30 °C for 6 h. The solvent was then discarded, and the remaining solid was dried in a fume hood for 3 h. Subsequently, hemicellulose removal was carried out by treating the dried powder with 800 mL of 6% (w/v) potassium hydroxide (KOH) solution under continuous stirring at 250 rpm at 30 °C for 24 h. The mixture was separated by centrifugation at 5000 rpm for 3 min, and the supernatant was discarded. The solid residue was washed with distilled water (800 mL per wash) and centrifuged at 500 rpm for 3 min at room temperature (25 °C); this washing step was repeated five times. Finally, delignification was carried out by bleaching the obtained solid with 200 mL of 10% (v/v) hydrogen peroxide at 80 °C under vigorous stirring at 250 rpm for 2 h, and the treatment was repeated twice. The resulting cellulose was washed with distilled water until the pH of the wash water approached neutrality, then dried in a hot-air oven at 55 °C for 48 h and stored in a desiccator until further use.

The synthesis of CMC from OPTS-derived cellulose was carried out *via* two main steps, namely alkalinization and etherification, following a previously reported method under optimized conditions (alkali concentration, etherification temperature and reaction time) with slight modifications.^33^ During alkalinization, 5 g of cellulose was placed in a beaker, and 20 mL of 30% (w/v) sodium hydroxide (NaOH) solution, corresponding to 6.0 g NaOH and a NaOH-to-cellulose mass ratio of 1.20 : 1, was added dropwise over 30 min. Subsequently, 150 mL of isopropanol was added, and the mixture was stirred at 250 rpm and 30 °C for 2 h. For the etherification step, sodium monochloroacetate (SMCA; 5 g), corresponding to an SMCA-to-cellulose mass ratio of 1 : 1, dissolved in 50 mL of isopropanol was added, and the reaction mixture was stirred at 50 °C for 3 h. After completion of the reaction, the liquid phase was discarded, and the resulting solid (CMC) was dispersed in 150 mL of absolute methanol. The dispersion was adjusted to pH 7 using glacial acetic acid and subsequently filtered through a Büchner funnel. The collected CMC was washed three times with 50 mL of 70% (v/v) ethanol to remove residual salts, followed by rinsing with 100 mL of absolute ethanol to eliminate residual moisture. The product was then dried in a hot-air oven at 55 °C for 24 h, ground, passed through a 60-mesh sieve, and stored in a desiccator. The CMC samples prepared from OPTS obtained from the bottom and middle sections were designated as CMC-b and CMC-m, respectively. The yields of OPTS cellulose and CMC were calculated using the following equations:1
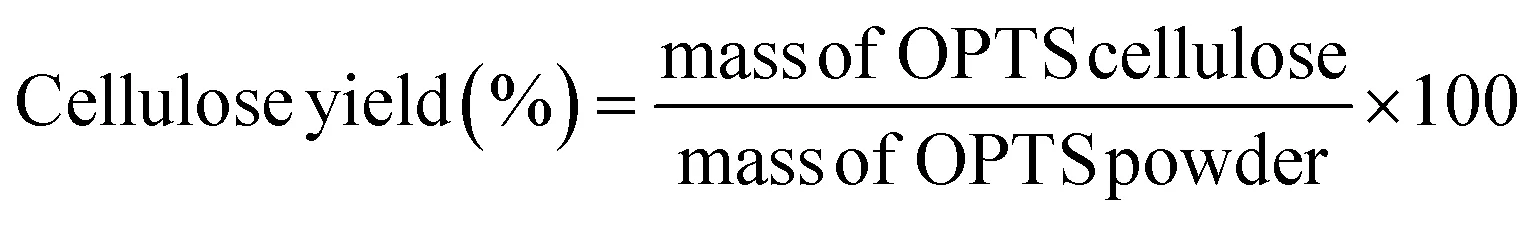
2
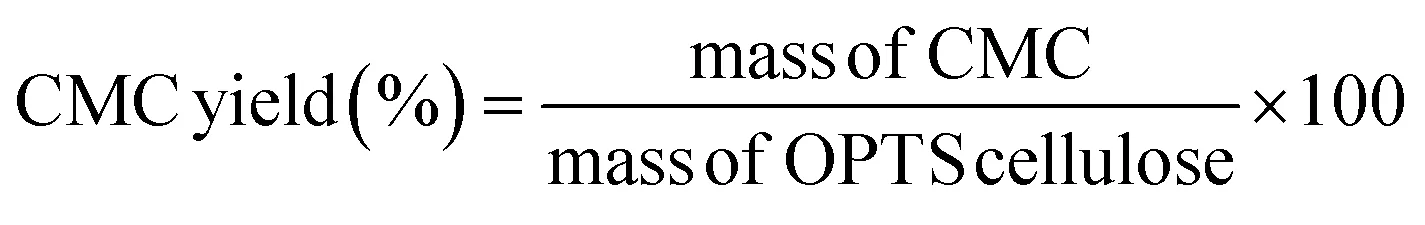


### Characterization of synthesized CMC

#### Fourier transform infrared (FTIR) spectroscopy analysis

FTIR was employed to investigate changes in functional groups during OPTS cellulose extraction and subsequent CMC synthesis. The powdered samples were compressed into transparent discs using a hydraulic press and analyzed using an FTIR spectrometer (Tensor 27; Bruker, Ettlingen, Germany) equipped with an attenuated total reflectance (ATR) accessory. Spectra were recorded over a wavenumber range of 4000–500 cm^−1^ with a resolution of 16 scans.

#### Differential scanning calorimetry (DSC) analysis

DSC was employed to confirm the successful synthesis of CMC. The synthesized CMC samples were analyzed in comparison with OPTS, extracted cellulose, and commercial CMC (CMC®) using a differential scanning calorimeter (DSC 6000; PerkinElmer, Waltham, MA, USA). Approximately 2 mg of each powdered sample was placed in an aluminum pan, hermetically sealed, and analyzed over a temperature range of 0–300 °C at a heating rate of 5 °C min^−1^ under a nitrogen flow rate of 20 mL min^−1^.

#### Scanning electron microscopy (SEM) analysis

The surface morphology of the prepared samples was examined using SEM (Zeiss Merlin FEG-SEM; Carl Zeiss, Oberkochen, Germany). Prior to analysis, the samples were sputter-coated with a thin layer of gold using a sputter coater (108 Auto; Cressington, Watford, UK) to enhance conductivity. Particle dimensions were estimated from the SEM micrographs by measuring the long axis and the width of individual fragments or particles. The width was measured approximately perpendicular to the long axis. Measurements were taken from five different fragments or particles for each sample (*n* = 5), and the results were reported as mean ± standard deviation (SD).

### Evaluation of physicochemical properties of synthesized CMC

#### Determination of degree of substitution (DS)

DS of the synthesized CMC was determined by acid–base titration using the residual titration method adapted from ASTM D1439-15.^34^ Briefly, 1 g of CMC was mixed with 50 mL of 95% (v/v) ethanol and stirred at room temperature (25 °C) for 5 min. Subsequently, 5 mL of 2 M nitric acid was added, and the mixture was stirred for an additional 10 min, followed by boiling at 100 °C for 30 min. The resulting sample was washed with 100 mL of 95% ethanol, rinsed with 100 mL of absolute ethanol, and dried in a hot-air oven at 70 °C for 3 h. A dried sample (0.5 g) was then dispersed in 100 mL of deionized water, and 25 mL of 0.5 M sodium hydroxide (NaOH) solution was added. The mixture was boiled at 100 °C for 30 min and allowed to cool to room temperature. Two drops of phenolphthalein were added as an indicator, and the solution was titrated with 0.3 M hydrochloric acid (HCl) until the colour changed from pink to colourless. The DS was calculated using the following equations:3
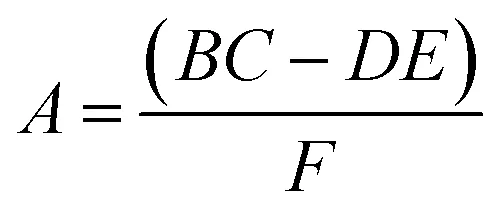
4
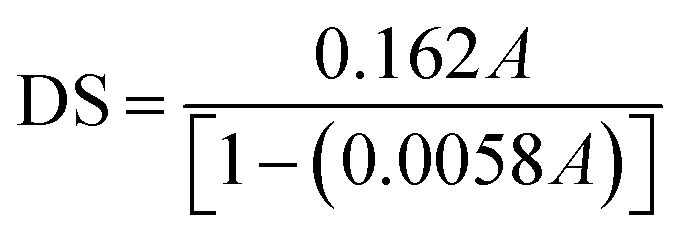
where *A* is the milli equivalents of NaOH consumed by the sample per gram of sample (meq per g); *B* is the volume of NaOH added (mL); *C* is the molarity of NaOH (M); *D* is the volume of HCl used for back titration (mL); *E* is the molarity of HCl (M); and *F* is the mass of the dry CMC sample (g). DS represents the degree of substitution of CMC and the constants 0.162 and 0.0058 are derived from the molecular weight of the anhydroglucose unit of cellulose (162 g mol^−1^) and the net increase in molecular weight associated with each substituted carboxymethyl group (58 g mol^−1^), respectively. All experiments were performed in triplicate.

#### Viscosity determination

A 1% (w/v) CMC solution was prepared in distilled water for viscosity measurements.^2^ The weighted CMC was dispersed in water and allowed to hydrate for 24 h to ensure complete swelling. The sample was then centrifuged at 3000 rpm to remove any undissolved matter, and the clear supernatant was used for viscosity determination. Viscosity was measured using a Brookfield viscometer (LVT model; Ametek (Thailand) Co. Ltd, Thailand) equipped with a cup-and-bob set at 25 °C. Measurements were performed at a rotational speed of 2.8 rpm. This speed generated a torque value close to 100% (>95%) for the reference CMC® solution and was therefore maintained constant for all samples to ensure valid comparative viscosity measurements. All experiments were performed in triplicate.

#### Determination of moisture content

The moisture content of the CMC samples was determined using a moisture analyser (HR83; Mettler-Toledo, Greifensee, Switzerland) with approximately 1 g of sample. All experiments were performed in triplicate.

#### pH determination

The pH of the CMC samples was measured using a pH meter at room temperature (25 °C). A 1% (w/v) CMC solution prepared in distilled water for viscosity measurements was also used for pH determination. All experiments were performed in triplicate.

### Preparation of acyclovir-loaded buccal films

To evaluate the potential pharmaceutical application of OPTS-derived CMC, the synthesized CMC was used to prepare acyclovir-loaded buccal films and compared with films prepared using commercial CMC (CMC®). The optimized formulation employed in this study was adopted from a previous study with slight modification,^35^ and is presented in Table 1. OPTS-derived CMC samples (CMC-b and CMC-m) were evaluated comparatively alongside CMC®. CMC was dispersed in purified water and gently stirred at room temperature (25 °C) for 12 h to ensure complete swelling. Acyclovir was then added and mixed until a homogeneous dispersion was obtained, with mild heating at 45 °C applied to facilitate dissolution. Polyvinylpyrrolidone K30 (PVP K30) and polyethylene glycol 400 (PEG 400) were added and mixed until a homogeneous mixture was obtained. The resulting mixture was cast into Teflon® Petri dishes (10 cm in diameter) and dried in a hot-air oven at 45 °C for 24 h to obtain acyclovir-loaded buccal films. The prepared films were stored in an airtight container until further evaluation.

**Table 1:** Composition of acyclovir-loaded buccal film formulations

Component	Content	Function
Acyclovir	63 mg	Active ingredient
CMC	600 mg	Viscosity-increasing and mucoadhesive agents
PVP K30	6 mg	Binder
PEG 400	15 mL	Plasticizer
Purified water	30 mL	Vehicle

### Characterization of acyclovir-loaded buccal films

#### Appearance, thickness, and weight uniformity

The general appearance of the buccal films was evaluated by visual inspection, and their texture and colour were recorded. Film thickness was measured at three different positions on each buccal film using a digital micrometer (vernier caliper). The three measurements were used to calculate the mean thickness and standard deviation for each film. For weight uniformity determination, three film specimens measuring 2 cm × 2 cm were cut from the buccal films and weighed individually using an analytical balance. The weight uniformity was reported as mean ± standard deviation (SD) (*n* = 3).

#### pH determination

The pH of the buccal films was determined by incubating 2 cm × 2 cm film samples in 2 mL of distilled water at room temperature (25 °C) for 2 h. The pH of the resulting solution was then measured using a calibrated pH meter. All experiments were performed in triplicate.

#### Determination of mucoadhesive force

The mucoadhesive force of the acyclovir-loaded buccal films was evaluated using porcine buccal mucosa. The experimental protocol involving the use of porcine buccal mucosa was approved by the Institutional Animal Care and Use Committee, Walailak University (approval no. WU-ACUC-67087). Buccal mucosa was obtained from 3-month-old pigs from a local slaughterhouse, carefully excised into thin sheets using surgical scissors, and rinsed with simulated salivary fluid (SSF), composed of 2.382 g disodium hydrogen phosphate, 0.19 g potassium dihydrogen phosphate, and 8 g sodium chloride per liter of distilled water. The pH of the SSF was adjusted to 6.8 using phosphoric acid prior to use. Mucoadhesive force was measured using a modified two-arm balance.^34^ Buccal mucosa sheets (3 cm × 3 cm) were secured over the mouth of a vial containing SSF using a rubber band, and the vial was placed in a beaker filled with SSF maintained at 37 ± 1 °C. A buccal film (3 cm × 3 cm) was attached to the underside of a rubber stopper, which was connected to the balance *via* a string. The film was brought into contact with the mucosa surface, while the opposite pan of the balance was gradually loaded with water by manual addition until detachment occurred. The weight of water required to detach the film from the mucosa was recorded, and the corresponding mucoadhesive force was calculated using the following equations:5
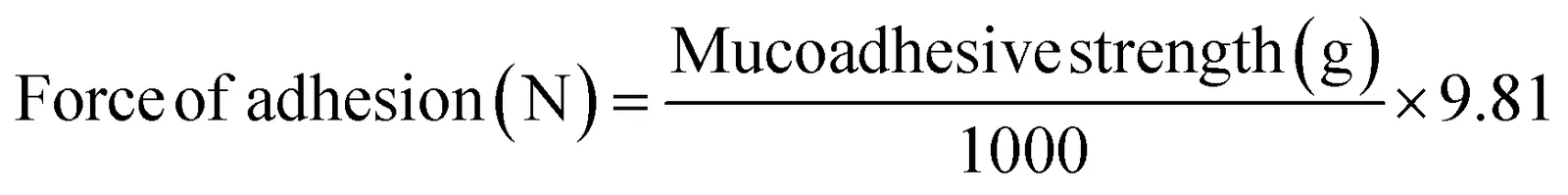
6
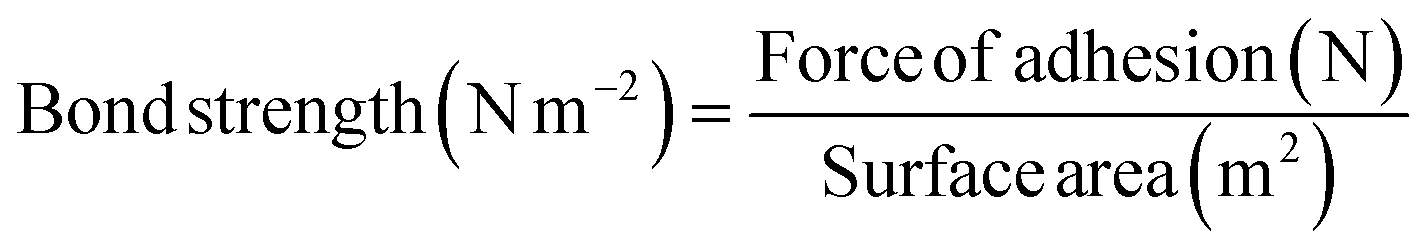


All experiments were performed in triplicate.

#### Determination of residence time

Residence time was determined using a modified United States Pharmacopeia (USP) disintegration apparatus.^36^ Briefly, 800 mL of SSF was used as the medium and maintained at 37 ± 1 °C in a beaker. Buccal mucosa was excised into 2 cm × 3 cm sections and mounted onto glass slides, which were subsequently secured to the disintegration basket. Prior to film application, the mucosal surface was moistened with 2 mL of SSF, and a 1 cm × 1 cm buccal film was placed onto the tissue. The disintegration apparatus was then operated, allowing the basket to move vertically while ensuring that the buccal film remained fully immersed in the medium throughout the test. The residence time was defined as the duration for which the buccal film remained adhered to the buccal mucosa before detachment. All experiments were performed in triplicate.

#### Drug content determination and *in vitro* drug release study

The acyclovir content in the buccal films was determined by cutting the films into 1 cm × 1 cm sections and placing each section in 2 mL of dimethyl sulfoxide (DMSO). Drug extraction was performed by sonication at room temperature (25 °C) for 15 min. All experiments were performed in triplicate. The extracted acyclovir was quantified using high-performance liquid chromatography (HPLC; Thermo Scientific, Waltham, MA, USA) equipped with a C18 analytical column (4.6 mm × 25 cm, 10 µm; Agilent, USA). Chromatographic analysis was conducted in accordance with the USP 2020 monograph for acyclovir.^37^ The mobile phase consisted of glacial acetic acid in water (1 : 1000, v/v), delivered at a flow rate of 3 mL min^−1^. The injection volume was 20 µL, and UV detection was performed at 254 nm.

The *in vitro* drug release of acyclovir from the buccal films was evaluated according to a previously reported method,^38^ with slight modifications, using a Franz diffusion cell with an effective diffusion area of 1.78 cm^2^ and a receptor compartment volume of 12 mL. Buccal films were cut into approximately 2 cm × 2 cm sections and placed on a cellulose acetate membrane (pore size 0.45 µm), followed by placement of a cellulose filter paper (pore size 11 µm; Whatman no. 1) on top of the film. The diffusion cell was assembled, and SSF was used as the receptor medium. The solubility of acyclovir in SSF was determined to be 2.11 ± 0.52 mg mL^−1^, confirming that sink conditions were maintained throughout the release study. To simulate buccal conditions, 400 µL of SSF was added to the donor compartment. The receptor medium was maintained at 37 °C throughout the experiment. Each formulation was evaluated in triplicate. Samples were withdrawn at predetermined time intervals (15 and 30 min, and 1, 2, 4, 6, and 12 h) and analyzed for acyclovir content using the HPLC method described above. The 12-h study period was selected to characterize the cumulative drug-release behavior of the films under standardized *in vitro* conditions and to facilitate comparison among the different CMC-based formulations.

The kinetics of drug release were further evaluated by fitting the *in vitro* release data to zero-order, first-order, Higuchi, and Korsmeyer–Peppas models. The release profiles were plotted according to each model as follows: zero-order (cumulative amount of drug released *vs.* time), first-order (log cumulative percentage of drug remaining *vs.* time), Higuchi (cumulative percentage of drug released *vs.* square root of time), and Korsmeyer–Peppas (log cumulative percentage of drug released *vs.* log time).^39^ Linear regression analysis was performed for each model, and the correlation coefficient (*R*^2^) was used to assess the goodness of fit. The model exhibiting the highest *R*^2^ value (*i.e.*, closest to 1) was considered to best describe the drug release kinetics. For the Korsmeyer–Peppas model, the release exponent (*n*) was determined from the slope of the linear regression and used to characterize the mechanism of drug release. Values of *n* ≤ 0.5 indicate Fickian diffusion, 0.5 < *n* < 1.0 indicate anomalous (non-Fickian) transport, and *n* ≥ 1.0 suggest case II transport or erosion-controlled release.^39^

Furthermore, the similarity factor (*f*_2_) was calculated to compare the release profiles of films prepared with CMC-b and CMC-m against those prepared with CMC®. The *f*_2_ value was determined using the standard equation recommended for dissolution profile comparison, where values between 50 and 100 indicate similarity between the two release profiles.^40^

#### *Ex vivo* buccal permeation study

The buccal permeation of acyclovir from the acyclovir-loaded buccal films was evaluated according to a previously reported method,^38^ with slight modifications, using excised porcine buccal mucosa and a Franz diffusion cell. Buccal films were cut into approximately 2 cm × 2 cm sections and placed directly onto the porcine buccal mucosa, with a cellulose filter paper (Whatman no. 1) positioned on top of the film. The diffusion cell was then assembled, and phosphate-buffered saline (PBS, pH 7.4) was added to the receptor compartment (12 mL). The solubility of acyclovir in PBS was determined to be 1.98 ± 0.09 mg mL^−1^, confirming that sink conditions were maintained throughout the permeation study. To simulate buccal conditions, 400 µL of SSF was added to the donor compartment onto the filter paper. The receptor medium was maintained at 37 °C throughout the experiment. Each formulation was evaluated in triplicate. Samples were withdrawn from the receptor compartment at predetermined time intervals (15 and 30 min, and 1, 2, 4, 6, and 12 h) and analyzed for acyclovir content using HPLC method described above. The permeation profiles and permeation parameters, including lag time, steady-state flux, and permeability coefficient, were subsequently determined. The steady-state flux was calculated from the slop of the linear portion of the permeation profile *vs.* time plot, normalized to the diffusion area. The permeability coefficient was calculated by dividing the flux by the initial acyclovir concentration in donor compartment.

## Results and discussion

### Cellulose extraction from OPTS and synthesis of CMC

The OPTS used in this study were obtained from the bottom and middle portions of the trunk, rather than the upper portion, due to the known variation in cellulose content along the trunk height. The upper portion contains a lower cellulose content compared to the middle and bottom sections, making it less suitable for cellulose extraction. Previous reports have indicated that the parenchyma tissue in the upper section contains relatively low cellulose content (approximately 8%), whereas the middle and bottom sections contain more than 30% cellulose.^20^ In this study, the yields of cellulose extracted from OPTS-b and OPTS-m were 41.43 ± 0.25% and 28.15 ± 0.33%, respectively (*n* = 3 each). The cellulose yield from OPTS-b falls within the range reported for oil palm-derived biomass (30–50%),^13^ whereas the yield from OPTS-m is comparatively lower. These results indicate a substantial difference in cellulose availability between the two sections of OPTS. This variation may be attributed to differences in intrinsic composition, including lignin and hemicellulose content, as well as the presence of other components such as starch, which is consistent with the morphological features observed in the SEM analysis.

The extracted cellulose from both OPTS-b and OPTS-m appeared off-white, with slight differences in coloration. Cellulose derived from the bottom section exhibited a slightly darker shade than that from the middle section, as shown in Fig. 1. This variation was also observed in the corresponding CMC samples, where CMC derived from the bottom section (CMC-b) appeared slightly darker than that obtained from the middle section (CMC-m), and both were marginally darker than commercial CMC (CMC®). This difference in coloration may be attributed to the age-related characteristics of the oil palm trunk, as the lower portions are generally older and tend to exhibit darker coloration than the upper portions. The colour of CMC could potentially be further lightened by increasing the number of bleaching cycles or extending the bleaching time,^41^ or by employing more intensive bleaching conditions during cellulose extraction, such as treatment with sodium hypochlorite instead of hydrogen peroxide,^42–44^ in order to enhance delignification efficiency. However, from safety and environmental perspectives, hydrogen peroxide is considered a greener and more sustainable reagent for cellulose preparation.^45^ The use of a common bleaching protocol, double bleaching with 10% hydrogen peroxide, as applied in this study, can therefore be considered acceptable, as it produced an off-white CMC rather than a completely white product; nevertheless, the material still complied with the specification for pharmaceutical-grade CMC, which describes the material as a “white or almost white” powder.^2^ However, the potential influence of these slight colour differences on key properties of CMC, particularly its toxicity profiles, should be further confirmed in future studies.

**Fig. 1:**
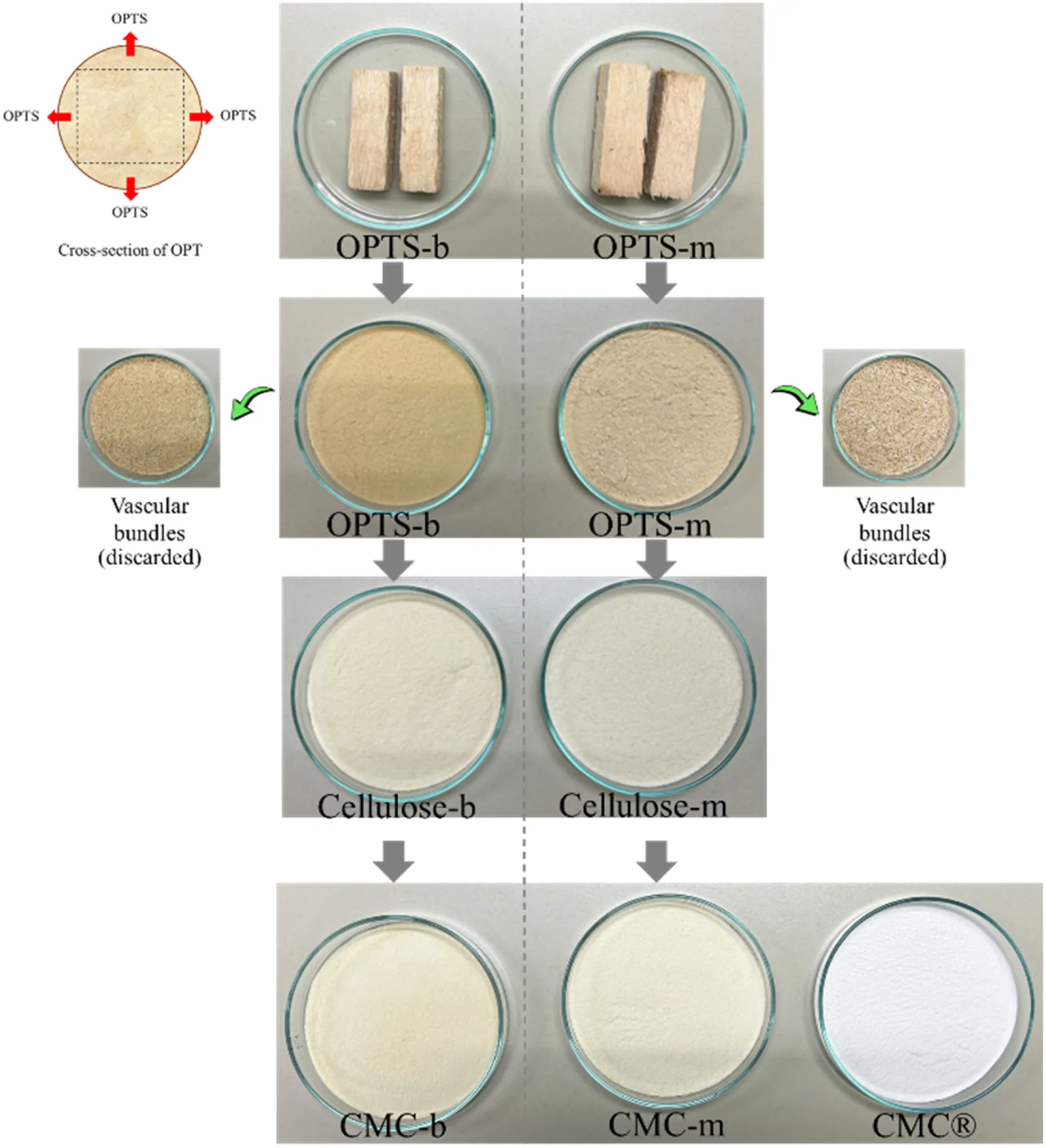
Appearance of OPTS obtained from the bottom (OPTS-b) and middle (OPTS-m) sections of the OPT, along with their corresponding cellulose products (Cellulose-b and Cellulose-m) and CMC products (CMC-b and CMC-m), compared with the commercial CMC®.

The yields of synthesized CMC-b and CMC-m were 119.41 ± 0.36% and 120.06 ± 0.32%, respectively. Despite the lower cellulose yield from OPTS-m, the subsequent carboxymethylation reaction produced comparably high yields for both samples, indicating that the reaction proceeded efficiently regardless of the cellulose source. These findings suggest that although OPTS-b is a more favourable raw material for cellulose extraction in terms of yield, celluloses derived from both OPTS-b and OPTS-m are suitable precursors for CMC production, exhibiting high conversion efficiency.

### Characterization of synthesized CMC

#### FTIR analysis

FTIR was employed to monitor the chemical composition and structural evolution of OPTS during purification and subsequent derivatization. The FTIR spectra of OPTS powder exhibited characteristic absorption bands of cellulose, along with signals corresponding to hemicellulose and lignin components (Fig. 2). The cellulose backbone was identified by a broad O–H stretching band at approximately 3300 cm^−1^, C–H stretching at approximately 2900 cm^−1^, and asymmetric stretching of the C–O–C glycosidic linkages at approximately 1100 cm^−1^.^46^ In addition, absorption bands at approximately 1700 cm^−1^ were attributed to C=O stretching of acetyl and uronic ester groups in hemicellulose (xylan),^47^ while bands near 1200 cm^−1^ corresponded to C–O stretching vibrations associated with xylan and lignin.^48^ Following alkali treatment with NaOH, the disappearance of peaks around 1700 cm^−1^ and 1200 cm^−1^ indicates the effective removal of hemicellulose and lignin. This observation highlights the critical role of the alkaline treatment in cellulose purification, to cleave ester linkages.^49^ Subsequent bleaching with hydrogen peroxide further enhanced the intensity and sharpness of the cellulose-associated peaks, reflecting increased purity and exposure of hydroxyl groups due to the removal of residual lignin and chromophoric impurities, as illustrated in Fig. 2A-d and B-j.

**Fig. 2:**
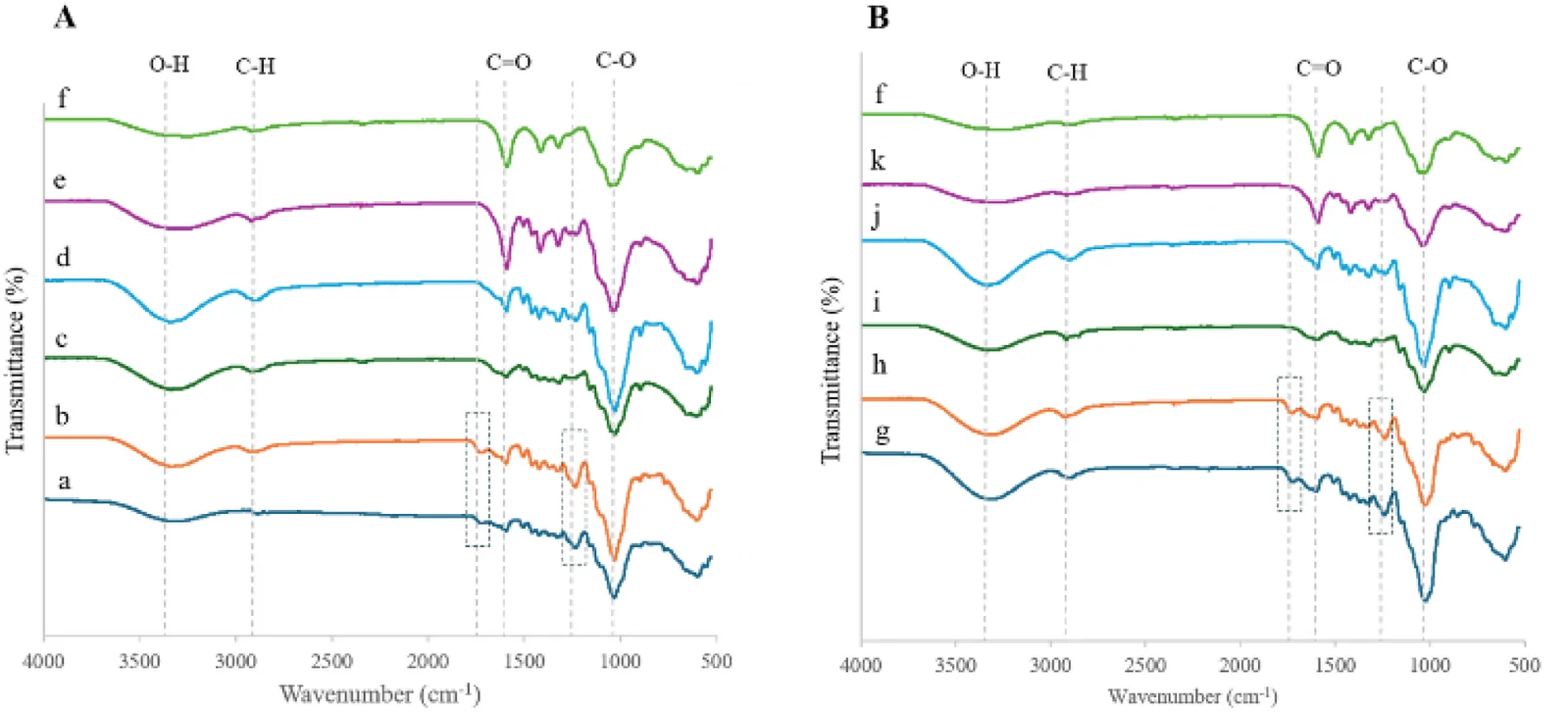
FTIR spectra of materials obtained during cellulose extraction and CMC synthesis from (A) OPTS-b and (B) OPTS-m, where (a and g) represent OPTS powder; (b and h) represent dewaxed OPTS; (c and i) represent OPTS after hemicellulose removal; (d and j) represent cellulose after delignification/bleaching; e and k represent synthesized CMC; and f represents commercial CMC (CMC®).

Upon conversion of cellulose into CMC, notable changes in the FTIR spectra were observed. In addition to the characteristic cellulose bands of cellulose, a new absorption band emerged in the range of approximately 1600–1700 cm^−1^, corresponding to the C=O stretching vibration of carboxymethyl groups. This feature is a key indicator of successful substitution. Other characteristic bands of CMC were also identified, including O–H stretching at around 3300 cm^−1^, C–H stretching near 2900 cm^−1^, and C–O–C ether bond vibrations at approximately 1100 cm^−1^.^50^ Furthermore, the FTIR spectra of the synthesized products (Fig. 2A-e and B-k) closely resembled that of commercial CMC® (Fig. 2A-f and B-f). Collectively, these spectral features confirm the successful etherification of cellulose into CMC.

#### DSC analysis

DSC was employed to assess chemical and structural modifications in the extracted and synthesized materials. The corresponding thermograms are presented in Fig. 3A–C. OPTS-b and OPTS-m exhibited similar thermograms, each showing two prominent endothermic peaks at approximately 102 °C and 275 °C, followed by the onset of thermal decomposition near 300 °C. These multiple thermal transitions reflect the heterogeneous composition of OPTS. The major endothermic peak at approximately 103 °C is attributed to the removal of bound water associated with cellulose.^51^ The consistent peak position observed for cellulose-b and cellulose-m indicates similar moisture-binding environments, which is in agreement with FTIR results showing comparable intensity and sharpness of the cellulose-associated bonds after purification.

**Fig. 3:**
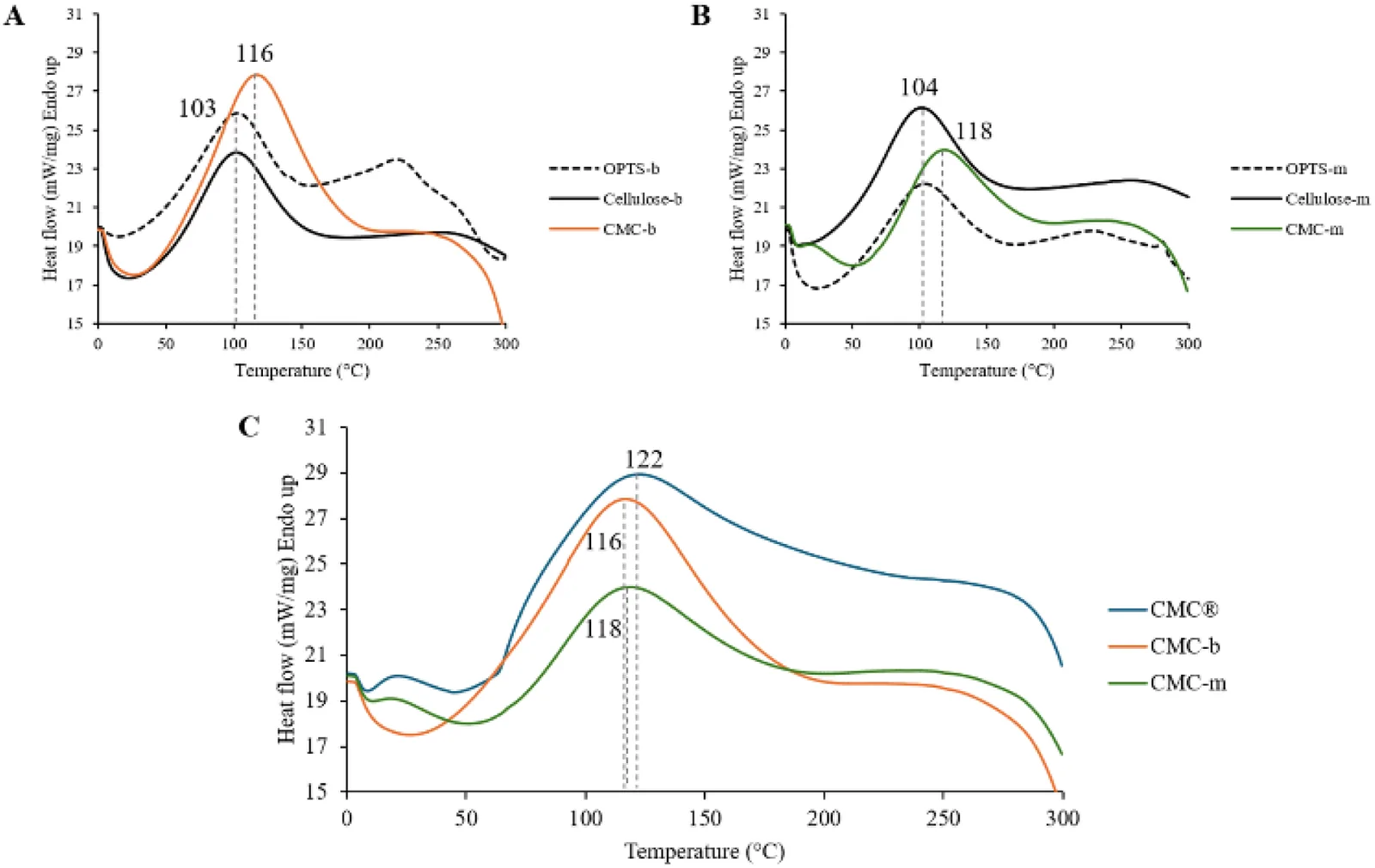
DSC thermograms of OPTS, extracted cellulose, and synthesized CMC obtained from (A) OPTS-b and (B) OPTS-m, compared with (C) commercial CMC (CMC®).

Following etherification to CMC, this endothermic peak shifted to higher temperatures (116 °C and 118 °C for CMC-b and CMC-m, respectively). This shift can be directly correlated with FTIR findings, which confirmed the introduction of carboxymethyl groups into the cellulose backbone.^52^ These groups increase polymer polarity and promote stronger interactions with water through hydrogen bonding and ion–dipole interactions.^53^ Consequently, greater thermal energy is required to remove the more tightly bound water, resulting in an elevated endothermic peak temperature. In addition, the chemical modification disrupts the native crystalline structure of cellulose, yielding a more amorphous matrix with increased accessibility to water molecules (crystallinity data for the synthesized CMCs are provided in the SI, Fig. S1). Despite enhanced water uptake, the strength of polymer–water interactions dominates, leading to higher desorption temperatures. When compared with commercial CMC (CMC®), the slightly lower transition temperatures observed for OPTS-derived CMC suggest a lower degree of substitution (DS), which corresponds to fewer carboxymethyl groups and weaker overall polymer–water interactions.

#### SEM analysis

SEM was employed to investigate the surface morphology and microstructural changes of OPTS, extracted cellulose, and synthesized CMC (Fig. 4). All samples were examined at the same magnification (100×) to enable direct comparison. OPTS-b exhibited relatively flattened structures composed of small fragments (Fig. 4A), whereas OPTS-m showed a more porous morphology (Fig. 4B). Notably, OPTS-m contained a large number of spherical starch granules, which were rarely observed in OPTS-b, indicating compositional differences between two regions.^54^ The cellulose extracted from OPTS-b (Fig. 4C) and OPTS-m (Fig. 4D) displayed similar morphologies, characterized by small, flattened, and densely aggregated particles. The absence of starch granules in these samples further confirms the effectiveness of the extraction process, particularly for OPTS-m, which initially contained higher starch content, and explains its comparatively lower cellulose yield.

**Fig. 4:**
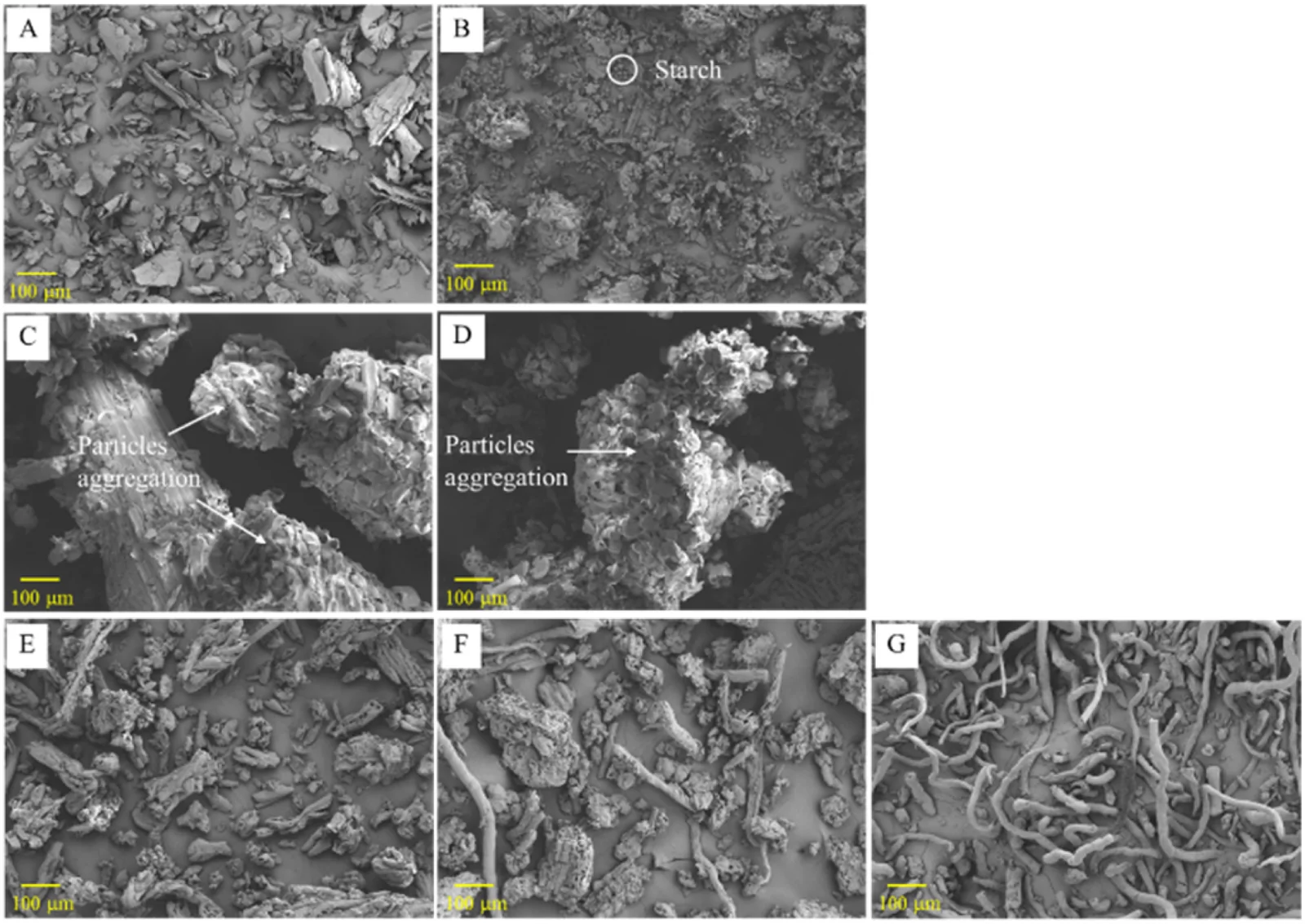
SEM micrographs of (A) OPTS-b powder, (B) OPTS-m powder, (C) cellulose-b, (D) cellulose-m, (E) CMC-b, (F) CMC-m, and (G) commercial CMC (CMC®).

Following etherification, CMC synthesized from OPTS-b (CMC-b; Fig. 4E) and OPTS-m (CMC-m; Fig. 4F) exhibited rougher surface textures and smaller particle sizes relative to commercial CMC (CMC®; Fig. 4G). The mean long-axis lengths of CMC-b, CMC-m, and CMC® were 105.04 ± 22.84 µm, 150.10 ± 54.74 µm, and 171.10 ± 12.26 µm, respectively, while the corresponding mean particle widths were 30.86 ± 2.59 µm, 45.71 ± 6.87 µm, and 25.20 ± 2.85 µm, respectively. A total of 5 particles per sample were analyzed. Although CMC-b and CMC-m showed shorter long-axis dimensions than CMC®, their greater widths indicate differences in particle morphology and dimensional characteristics. The increased surface roughness and fragmentation observed in CMC samples reflect partial disruption of the cellulose structure during alkalization and etherification. However, the persistence of aggregated features suggests that substitution as not extensive enough to fully disrupt intermolecular interactions or convert the structure into a highly swollen and homogeneous morphology. These morphological changes reflect the impact of etherification and subsequent processing on particle structure, which may influence the physicochemical and functional properties of the resulting materials.

### Physicochemical properties of synthesized CMC

#### Degree of substitution (DS) analysis

The DS of CMC represents the average number of hydroxyl groups on each anhydroglucose unit of cellulose that are replaced by carboxymethyl groups. In this study, the DS values of CMC-b and CMC-m were in the range of 0.4–0.5 (Table 2), which falls within the typical range reported for commercial CMC used in general applications (DS 0.4–1.5).^55^ No significant difference in DS was observed between the two samples, indicating that the anatomical origin of the oil palm trunk (bottom *vs.* middle section) had a negligible effect on the extent of substitution under the identical synthesis conditions employed. This may be attributed to the comparable chemical composition and reactivity of cellulose obtained from different trunk regions. In contrast, the higher DS observed for commercial CMC (CMC®, DS ∼0.8) is likely associated with differences in raw material purity, cellulose accessibility, and optimized industrial processing conditions designed to enhance substitution efficiency and uniformity.^7,56^

**Table 2:** Physicochemical properties of CMC synthesized from OPTS-b and OPTS-m compared with commercial CMC (CMC®). Data are presented as mean ± standard deviation (*n* = 3)

Properties	CMC-b	CMC-m	CMC®
Degree of substitution (DS)	0.45 ± 0.02	0.43 ± 0.01	0.81 ± 0.01
Viscosity (cP)	59.29 ± 3.44	48.08 ± 1.72	1058.33 ± 11.00
Moisture content (%)	8.50 ± 0.20	7.80 ± 0.17	7.00 ± 0.20
pH	7.30 ± 0.10	7.40 ± 0.10	7.43 ± 0.06

Although the theoretical maximum DS is 3.0, corresponding to complete substitution of all three hydroxyl groups, such values are rarely achieved due to steric hindrance, strong intra- and intermolecular hydrogen bonding, and the limited accessibility of hydroxyl groups, particularly within the crystalline regions of cellulose.^57^ In the present study, the relatively moderate DS values can be attributed to structural constraints that limit the efficiency of the etherification reaction. This is consistent with the morphological features observed in SEM micrographs (Fig. 4C and D), where the extracted cellulose exhibited a dense, flattened, and highly aggregated structure with low porosity. Such compact morphology likely restricts the penetration of alkali and etherifying agents into the cellulose fibers, thereby limiting the accessibility of reactive hydroxyl groups. As a result, the substitution reaction is expected to occur predominantly at the more accessible surface regions, leading to partial substitution and moderate DS values. Similar observations have been reported in previous studies, where compact cellulose structures and diffusion limitations in heterogeneous systems were shown to significantly reduce substitution efficiency.^7^

From an application perspective, moderate DS values are often desirable, as they provide a balance between water solubility, viscosity, and mechanical performance.^58^ Therefore, the synthesized CMC in this study is expected to exhibit adequate functionalization for water solubility while retaining sufficient structural integrity of the cellulose backbone, which is advantageous for applications such as film formation and drug delivery systems.

#### Viscosity analysis

Viscosity is a key functional property of CMC, directly influencing its applicability in drug delivery systems through its effects on solution rheology, drug diffusion, and mucoadhesive performance. In this study, the viscosities of the synthesized CMC samples (CMC-b and CMC-m), measured at 1% (w/v), ranged from approximately 45 to 60 cP (Table 2), indicating a low-viscosity grade.^2^ In contrast, the commercial CMC (CMC®) exhibited a markedly higher viscosity of approximately 1000 cP under the same conditions. This pronounced difference in viscosity can be partially interpreted in relation to the DS. The synthesized CMC samples exhibited moderate DS values (0.4–0.5), which are sufficient substitution to promote water solubility through the introduction of hydrophilic carboxymethyl groups; however, such DS levels do not necessarily result in high viscosity. Although viscosity is often correlated with DS, it is more strongly governed by molecular weight,^55^ polymer chain length, and the extent of intermolecular entanglement in solution.^59^

The reaction conditions used for CMC preparation may have contributed to the observed viscosity. The use of 30% NaOH at 50 °C for 3 h was selected to provide sufficiently strong alkaline conditions to promote cellulose swelling and activation, thereby facilitating reagent accessibility and the subsequent carboxymethylation reaction. This approach was intended to achieve an adequate DS, as insufficient alkalization may limit cellulose activation and result in lower substitution. However, alkaline treatment severity may have competing effects on cellulose structure. While strong alkaline conditions facilitate cellulose swelling and activation, prolonged exposure to a high NaOH concentration and elevated temperature may also increase the susceptibility of cellulose chains to partial degradation or depolymerization. Thus, the relatively intensive conditions used in this study may have contributed not only to cellulose activation and carboxymethylation but also to partial chain scission, potentially reducing the average polymer chain length.

The morphological characteristics observed from SEM provide insight into this behavior. The synthesized CMC samples displayed disrupted, fragmented, and less fibrous structures, in contrast to the more compact and unfirm morphology observed for CMC®. This structural disruption may reflect changes in the cellulose structure during the chemical conversion process and is consistent with the possibility of partial chain degradation. Although molecular weight and degree of polymerization were not directly determined in the present study, the markedly lower viscosity of CMC-b and CMC-m is consistent with a reduction in polymer chain length and molecular integrity. The resulting shorter polymer chains would provide fewer opportunities for intermolecular entanglement in aqueous solution, thereby reducing viscosity. The reduced chain length and viscosity may also affect the film-forming and mucoadhesive properties of the synthesized CMC. Shorter polymer chains provide fewer opportunities for intermolecular interactions and chain entanglement, potentially reducing polymer-network cohesion and mechanical integrity. This may contribute to the surface irregularities and cracks observed in the synthesized CMC films and may also help explain their substantially shorter mucoadhesive residence times compared with CMC®. In comparison, CMC®, despite having only a moderately higher DS (≈0.8), exhibited a significantly higher viscosity. This disparity supports that viscosity is not solely dependent on DS but is strongly influenced by polymer chain length and molecular integrity.

#### Moisture content and pH analysis

Moisture content and pH are important quality control parameters for CMC, particularly in pharmaceutical applications, as specified by pharmacopeial standards.^37^ The determination of moisture content is essential because excessive residual water can adversely affect the stability, flowability, and shelf life of the material, as well as promote microbial growth. In addition, moisture levels may influence the physicochemical properties of CMC, including its swelling behavior and viscosity in aqueous systems.^7^ Similarly, pH is a critical parameter because it affects the ionization of carboxymethyl groups, which in turn influences solubility, viscosity, and compatibility with active pharmaceutical ingredients (APIs). Maintaining an appropriate pH range is also important to minimize the risk of chemical degradation and to ensure safety and performance in drug delivery systems.^60^

In this study, both parameters (Table 2) were within acceptable limits specified by the USP. The moisture contents of the synthesized CMC samples were < 10%, while the pH values ranged between 6.5 and 8.5, complying with pharmacopeial specifications.^37^ These results indicate that the synthesized CMC meets the fundamental quality requirements for potential pharmaceutical applications.

### Characterization of acyclovir-loaded buccal films

#### General properties

Acyclovir-loaded buccal films were successfully prepared using the solvent casting and evaporation method. Films made from the synthesized CMC samples (CMC-b and CMC-m) exhibited a slightly off-white appearance, with CMC-b appearing marginally darker than CMC-m, whereas films prepared from commercial CMC (CMC®) were relatively transparent (Fig. 5). The observed colour differences correspond to the inherent colour of the respective CMC materials, reflecting variations in raw material origin and processing.

**Fig. 5:**
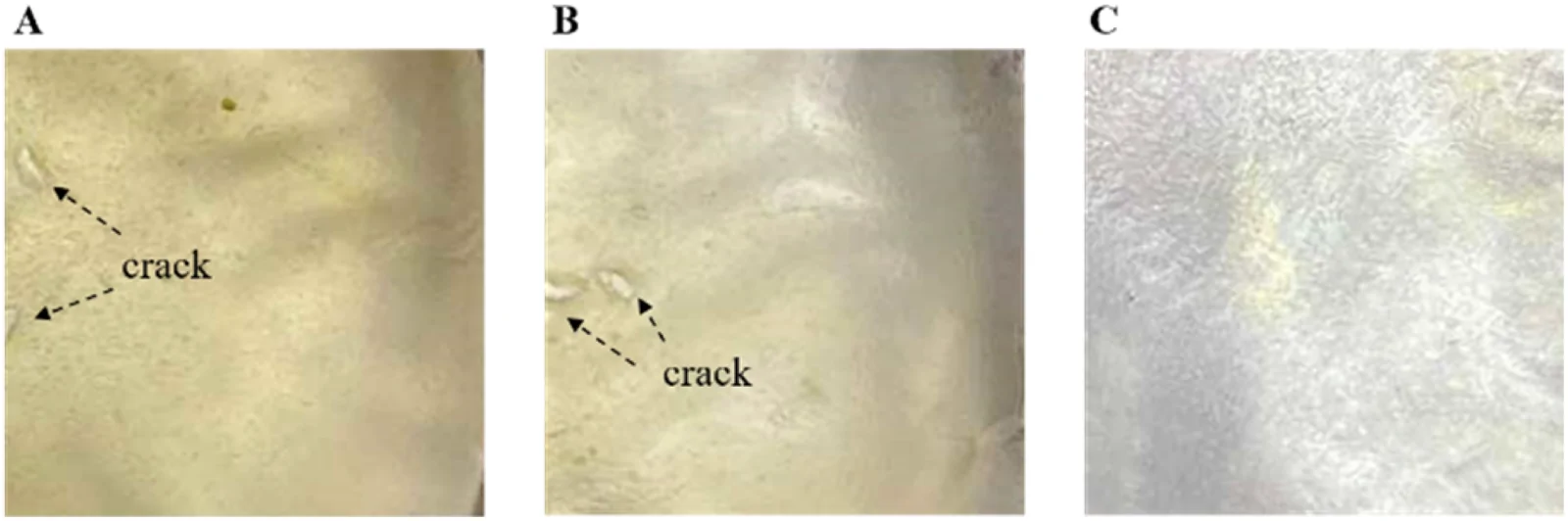
Visual appearance of acyclovir-loaded buccal films prepared using different types of CMC: (A) CMC-b, (B) CMC-m, and (C) commercial CMC®; arrows indicate surface cracks.

The surface morphology of films prepared from CMC-b and CMC-m exhibited localized cracks and surface irregularities, whereas films based on CMC® exhibited a more uniform and structurally intact surface. The lengths of two representative cracks were approximately 5.5 and 6.5 mm for CMC-b and 6 and 8 mm for CMC-m, respectively. This difference can be attributed to variations in viscosity and polymer chain characteristics, which influence film-forming behavior.^7^ Specifically, the lower viscosity of CMC-b and CMC-m suggests reduced molecular weight and diminished chain entanglement, resulting in weaker intermolecular cohesion during solvent evaporation, resulting in less cohesive film matrices. In contrast, the higher viscosity of CMC® indicates greater molecular weight and stronger intermolecular interactions, which promote the formation of a dense and homogeneous film network. Although these morphological features suggest differences in film integrity, their direct effects on mechanical properties could not be established because tensile properties were not evaluated in the present study. For subsequent drug content, drug release, permeation, and mucoadhesive evaluations, only visually intact film specimens without apparent cracks or other surface defects were selected. Further mechanical characterization, including tensile strength and elongation at break, would be useful for evaluating the structural integrity of these films.

Despite these morphological differences, the thickness and weight of all film formulations exhibited low standard deviations (Table 3), indicating good uniformity of casting and reproducibility of the preparation method. No significant differences were observed among films prepared from CMC-b, CMC-m, and CMC®. Similarly, the surface pH values of all buccal films were comparable across formulations, suggesting that the type of CMC did not markedly influence the acid–base characteristics of the final dosage form.

**Table 3:** General properties of acyclovir-loaded buccal films prepared using different types of CMC: CMC-b, CMC-m, and commercial CMC®. Data are presented as mean ± standard deviation (*n* = 3). RSD, relative standard deviation, was calculated as (SD/mean) × 100%

Properties	Type of CMC		
	CMC-b	CMC-m	CMC®
Thickness (mm)	0.24 ± 0.01	0.28 ± 0.01	0.23 ± 0.01
	RSD = 6.19%	RSD = 5.32%	RSD = 6.45%
Weight (mg cm^−2^)	0.38 ± 0.01	0.37 ± 0.01	0.37 ± 0.01
pH	7.06 ± 0.04	7.53 ± 0.09	7.76 ± 0.08

These results indicate that while the source and physicochemical properties of CMC notably affect film microstructure and mechanical integrity, they do not significantly alter the bulk physical parameters such as thickness, weight uniformity, and pH. This suggests that the solvent casting process was sufficiently robust to produce consistent films, although optimization of polymer properties, particularly molecular weight and viscosity, is essential to improve film quality and performance.

#### Mucoadhesive properties

The films prepared using commercial CMC (CMC®) exhibited the highest mucoadhesive force, whereas films containing the synthesized CMC samples (CMC-b and CMC-m) showed slightly lower values (Table 4). A similar trend was observed for bond strength. Although the mucoadhesive force of CMC-b films appeared lower than that of CMC-m films, the difference was not statistically significant (*p* > 0.05). Despite the substantially lower viscosity of the synthesized CMC samples (approximately 15-fold) lower than that of CMC®, the reduction in mucoadhesive force was relatively modest (less than 1.2-fold). This indicates that mucoadhesion is not governed solely by viscosity. While higher viscosity is generally associated with improved chain entanglement and mechanical interlocking with mucosal surfaces, other factors such as the availability of functional groups (*e.g.*, carboxymethyl groups), polymer flexibility, and the ability to form hydrogen bonds also play critical roles in adhesion.^7^ The moderate degree of substitution (DS 0.4–0.5) of CMC-b and CMC-m likely provides sufficient functional groups to maintain effective intermolecular interactions with mucin, thereby preserving mucoadhesive performance despite reduced molecular weight. It should be noted that manual addition of water as the counterweight without controlled flow or evaporation may have introduced some measurement variability.

**Table 4:** Performance-related properties of acyclovir-loaded buccal films prepared using different types of CMC: CMC-b, CMC-m, and commercial CMC®. Data are presented as mean ± standard deviation (*n* = 3)

Properties	Type of CMC		
	CMC-b	CMC-m	CMC®
Force of adhesion (N)	0.029 ± 0.001	0.029 ± 0.002	0.035 ± 0.001
Bond strength (N m^−2^)	286.70 ± 15.27	293.30 ± 25.16	346.70 ± 15.27
Residence time (min)	7.71 ± 0.29	7.27 ± 0.19	155 ± 9.16

In contrast, a pronounced difference was observed in retention time. Films containing CMC® exhibited retention times approximately 22-fold longer than those of films prepared from CMC-b or CMC-m. This substantial disparity suggests that retention time is more sensitive to polymer viscosity and mechanical integrity than the initial adhesive force. The higher molecular weight and viscosity of CMC® likely contribute to stronger cohesive strength within the polymer matrix and enhanced resistance to hydration, erosion, and detachment from the mucosal surface.^61^

These findings demonstrate that although the synthesized CMC samples can achieve initial mucoadhesive strength comparable to commercial CMC®, their lower viscosity and molecular integrity significantly compromise retention time. This indicates that the synthesized CMC films may be suitable for buccal drug delivery applications; however, their short mucoadhesive residence times may limit prolonged contact with the buccal mucosa. Therefore, although the synthesized CMC films showed favorable initial mucoadhesive properties, further formulation optimization is needed to extend residence time for applications requiring prolonged buccal retention.

#### Drug content and *in vitro* drug release behavior

The acyclovir content in the buccal films was determined to be 98.52 ± 1.44%, 98.85 ± 1.69%, and 98.26 ± 0.63% for films containing CMC-b, CMC-m, and CMC®, respectively. These values are close to 100%, indicating minimal drug loss during the film preparation process. The low standard deviations further suggested consistent acyclovir content among the film specimens analyzed. For drug content determination, specimens were cut from intact film areas without visible cracks. Therefore, the reported drug content represents the intact regions of the films and indicates consistent drug content in the specimens selected for analysis. The potential effect of localized surface cracks on drug distribution at or around the crack sites was not directly evaluated in this study.

The *in vitro* release profiles of acyclovir from the buccal films are presented in Fig. 6. All three formulations exhibited similar release patterns, characterized by a gradual increase in drug release over time, reaching a plateau within approximately 6 h. The comparable release behavior across different CMC types suggests that variations in polymer source, viscosity, and DS did not markedly affect the overall release profile under the studied conditions. This may be attributed to the hydrophilic nature of CMC, which facilitates rapid hydration and formation of a gel-like matrix, enabling consistent diffusion pathways for drug release. It should be noted that the 12-h release study was conducted to characterize the drug-release behavior of the film matrices under controlled *in vitro* conditions and was not intended to represent the duration of film residence on the buccal mucosa *in vivo*.

**Fig. 6:**
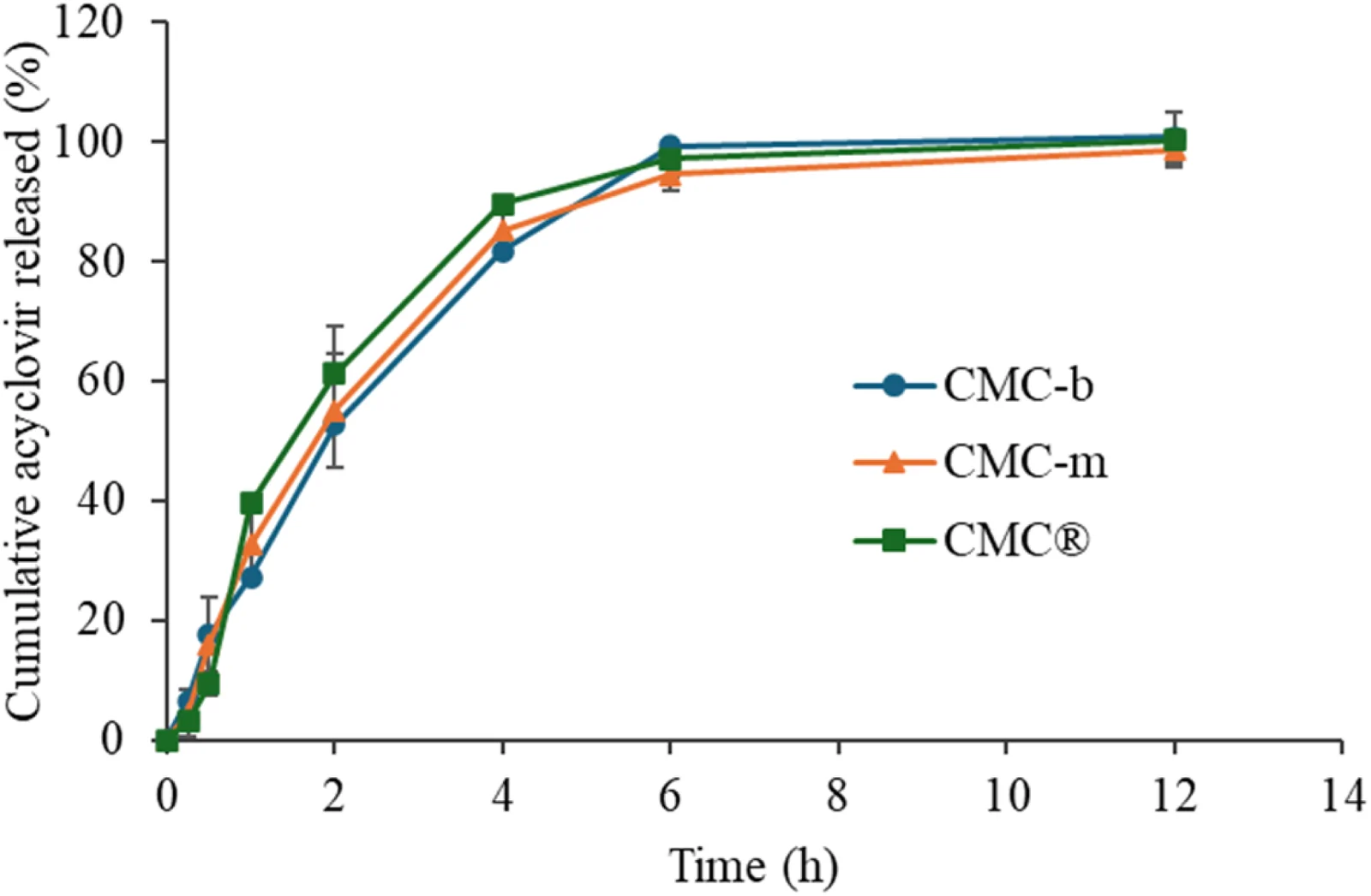
*In vitro* release profiles of acyclovir from buccal films prepared using different types of CMC: CMC-b, CMC-m, and commercial CMC®. Data are presented as mean ± standard deviation (*n* = 3).

Kinetic modelling revealed distinct release mechanisms among the formulations (drug release kinetic parameters obtained from various models, including zero-order, first-order, Higuchi, and Korsmeyer–Peppas, for acyclovir release from buccal films prepared using different types of CMC are presented in the SI, Table S1). The release profiles of CMC-b and CMC-m films were best described by the Kosmeyer–Peppas model, whereas the CMC® formulation followed the Higuchi and first-order models. The Korsmeyer–Peppas model is widely used to analyse drug release from polymeric systems when multiple mechanisms may be involved.^39^ The release exponent (*n*) values for CMC-b, CMC-m, and CMC® films were 0.902, 0.955, and 1.250, respectively. These *n* values provide insight into the underlying release mechanisms. For CMC-b and CMC-m films, *n* values between 0.5 and 1.0 indicate anomalous (non-Fickian) transport, suggesting that drug release is governed by a combination of diffusion and polymer relaxation or swelling.^39^ This behavior consists of the moderate viscosity and molecular weight of the synthesized CMC, which allows both water penetration and polymer chain mobility to contribute to drug release. In contrast, the *n* value greater than 1.0 for the CMC® film indicates super case II transport, which is associated with polymer relaxation- or erosion-controlled release mechanisms.^39^ This behavior can be attributed to the higher molecular weight and viscosity of commercial CMC, resulting in stronger intermolecular interactions and a more rigid polymer network. Upon hydration, polymer relaxation and swelling dominate the release process, leading to a distinct kinetic profile compared to the synthesized CMC films.

Despite these mechanistic differences, evaluation of the similarity factor (*f*_2_) demonstrated that the release profiles of films prepared from synthesized CMC were comparable to those of commercial CMC®. The *f*_2_ values for CMC-b and CMC-m relative to CMC® were 55.03 and 64.01, respectively, which fall within the acceptable similarity range of 50–100. These results indicate that both synthesized CMC samples provide drug release profiles comparable to commercial CMC. Although the macroscopic release profiles appear similar, the underlying release mechanisms differ depending on the physicochemical properties of the CMC used, particularly viscosity and molecular weight, which influence polymer relaxation and diffusion behavior.

#### *Ex vivo* buccal permeation study of acyclovir-loaded buccal films

The *ex vivo* buccal permeation profiles of acyclovir from buccal films prepared using different types of CMC, together with the permeation parameters including lag time, flux, and permeability coefficient, are shown in Fig. 7. All formulations exhibited a gradual increase in drug permeation across the buccal mucosa during the 12-h experimental period. The permeation profiles showed an initial lag phase prior to permeation, which may be attributed to hydration and swelling of the polymeric films, followed by diffusion of acyclovir through the swollen matrix, and subsequent partitioning of acyclovir into the buccal tissue. Films prepared from synthesized CMC (CMC-b and CMC-m) exhibited relatively shorter lag times (approximately 0.4–0.5 h) than the film prepared from commercial CMC® (approximately 0.9 h), although the differences were not statistically significant. The shorter lag time observed for the synthesized CMC-containing films may be related to their relatively lower viscosity and molecular weight, which could facilitate faster hydration and matrix swelling, thereby promoting earlier drug diffusion from the film into the buccal membrane. This observation corresponds well with the release study, in which CMC-b and CMC-m containing films exhibited anomalous (non-Fickian) transport behavior, reflecting the combined contribution of diffusion and polymer relaxation to drug release.

**Fig. 7:**
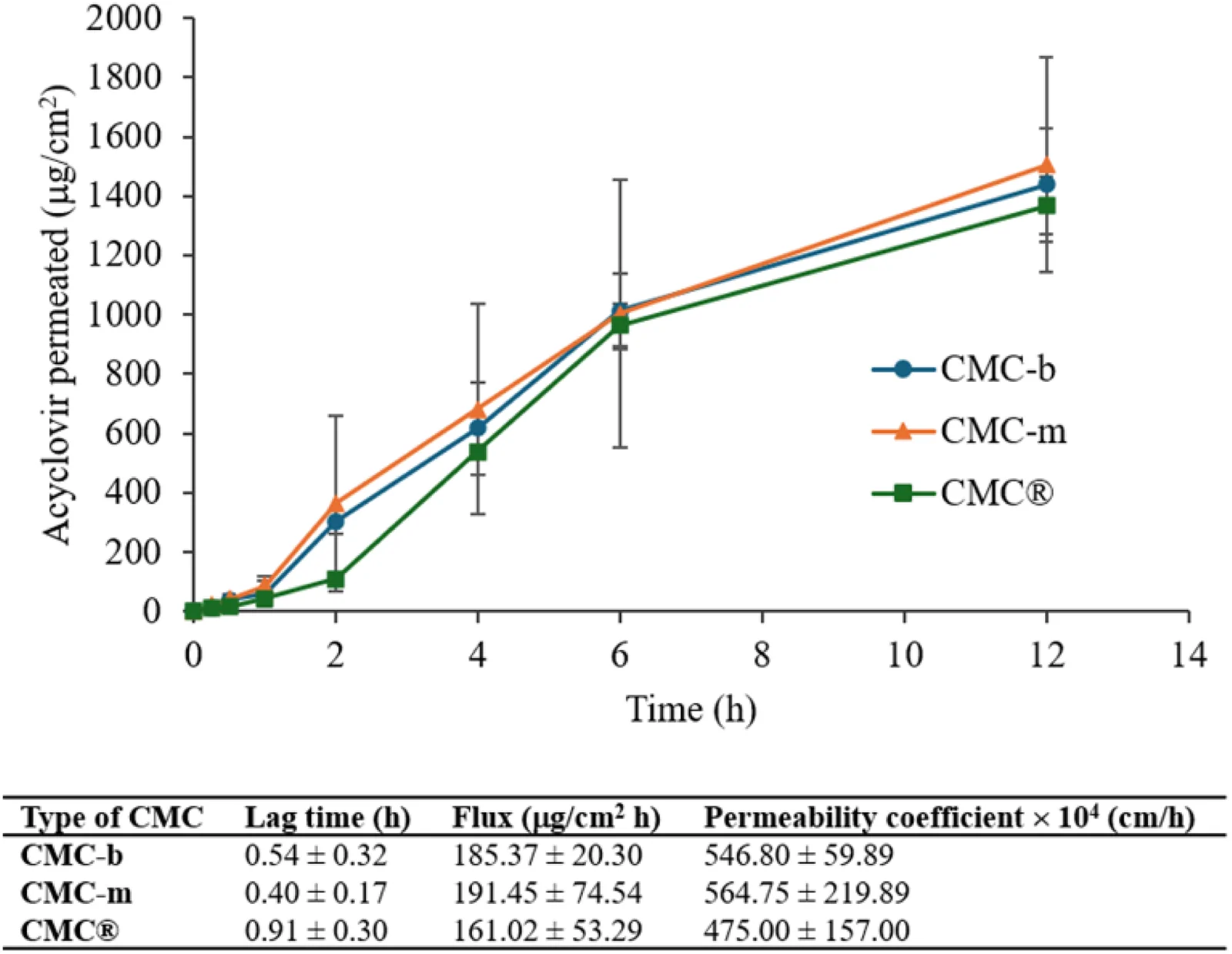
*Ex vivo* permeation profiles and permeation parameters of acyclovir from buccal films prepared using different types of CMC: CMC-b, CMC-m, and commercial CMC®. Data are presented as mean ± standard deviation (*n* = 3).

The flux values of films containing synthesized CMC were slightly higher than those of the commercial CMC®-containing film, with values of approximately 185, 190, and 160 µg cm^−2^ h^−1^ for CMC-b, CMC-m, and CMC® containing films, respectively. A similar trend was observed for the permeability coefficients, with values of approximately 545, 565, and 475 × 10^−4^ cm h^−1^ for CMC-b, CMC-m, and CMC® containing films, respectively. However, no statistically significant differences in flux or permeability coefficient were observed among the formulations (*p* > 0.05).

Although the synthesized CMC-containing films exhibited lower viscosity and distinct mucoadhesive properties compared with the commercial CMC®-containing film, these differences did not markedly influence the overall permeation of acyclovir across the buccal mucosa under the experimental conditions. The comparable permeation behavior among formulations may be attributed to the hydrophilic nature and relatively low membrane permeability of acyclovir, for which mucosal transport is primarily governed by drug diffusion through the hydrated mucosal tissue rather than by minor variations in polymer characteristics. However, considering the short mucoadhesive residence times of the synthesized CMC films, the observed *ex vivo* permeation profiles should be interpreted as reflecting the intrinsic release and permeation characteristics of the film matrices rather than prolonged retention and continuous drug delivery at the buccal site. Overall, the results demonstrate that synthesized CMC derived from both sources can provide buccal films with *in vitro* release and *ex vivo* permeation characteristics comparable to commercial CMC® under the experimental conditions, although further optimization of mucoadhesion and residence time is required for prolonged buccal delivery.

#### Biocompatibility considerations and study limitations

The chemical reagents used during the preparation of CMC, including toluene, ethanol, hydrogen peroxide, and sodium monochloroacetate, were subjected to subsequent purification and washing procedures before the CMC was used for film preparation. However, the present study did not directly determine residual processing reagents or evaluate the cytocompatibility of the resulting CMC-based buccal films. Therefore, biological safety for direct application to the oral mucosa cannot be conclusively established based solely on the physicochemical, drug-release, and *ex vivo* permeation results. Further studies using relevant oral mucosal cell models, such as oral keratinocytes or buccal epithelial cells, including cell-viability assays such as MTT, are warranted to confirm the cytocompatibility of the developed films before further consideration for clinical application.

## Conclusions

Carboxymethyl cellulose (CMC) was successfully synthesized from cellulose extracted from the bottom and middle sections of oil palm trunk slab (OPTS) waste using a conventional etherification method. The extracted celluloses from both OPTS regions were suitable precursors for CMC production, yielding products with moderate degrees of substitution (DS 0.4–0.5), acceptable moisture content and pH, and physicochemical characteristics comparable to those of commercial CMC®. FTIR and DSC analyses confirmed successful carboxymethylation, while SEM observations demonstrated structural modification of cellulose following etherification. Although the synthesized CMC exhibited substantially lower viscosity than commercial CMC®, the materials retained functional properties that supported their incorporation into buccal film formulations.

Acyclovir-loaded buccal films prepared from synthesized CMC were successfully fabricated using the solvent casting method and demonstrated satisfactory physicochemical characteristics, including uniform thickness, weight, surface pH, and drug content. The synthesized CMC-containing films exhibited mucoadhesive properties comparable to those of commercial CMC®-containing film in terms of adhesive force, although their residence times were considerably shorter, likely due to lower molecular weight and reduced cohesive strength of the polymer matrix. *In vitro* release and *ex vivo* permeation studies showed that films prepared from synthesized CMC provided drug release and permeation behaviors comparable to those of commercial CMC®, despite differences in release kinetics and polymer viscosity. The synthesized CMC-containing films exhibited anomalous (non-Fickian) transport behavior, indicating the combined contribution of diffusion and polymer relaxation during drug release.

Overall, the findings demonstrate that OPTS-derived CMC exhibited physicochemical and functional characteristics that support its potential use as a mucoadhesive and film-forming polymer in buccal drug delivery systems. However, the substantially shorter mucoadhesive residence time of the synthesized CMC-containing films may limit their application when prolonged buccal retention is required. This study highlights the potential of oil palm trunk slab waste as a sustainable and value-added source of pharmaceutical excipients. Although CMC synthesized from the bottom and middle sections of OPTS exhibited comparable physicochemical and functional properties, the bottom section provided a substantially higher cellulose yield, indicating its potential as a more productive raw material for CMC production. Further optimization of the synthesis and formulation processes, particularly to improve polymer viscosity and mucoadhesive retention, is warranted. In addition, direct biocompatibility evaluation of the developed buccal films and long-term stability are required before their pharmaceutical applicability can be further established.

## Author contributions

Chutima Jantarat: conceptualization, methodology, investigation, formal analysis, visualization, data curation, writing – original draft, writing – review & editing. Sirikanya Kaewpradit: methodology, investigation, formal analysis, visualization, data curation, writing – review & editing. Jiraporn Chingunpitak: methodology, formal analysis, visualization, writing – review & editing. Suthon Srivaro: methodology, formal analysis, visualization, writing – review & editing. All authors approved the final version of the manuscript before submission.

## Conflicts of interest

There are no conflicts to declare.

## Supplementary Material

RA-OLF-D6RA04937K-s001

## Data Availability

The supporting data has been provided as part of the supplementary information (SI). Supplementary information: XRD patterns of materials obtained during cellulose extraction and CMC synthesis, as well as drug release kinetic parameters derived from zero-order, first-order, Higuchi, and Korsmeyer–Peppas models for acyclovir release from buccal films containing different types of CMC. See DOI: https://doi.org/10.1039/d6ra04937k.
