# Supplementary material for: Carboxymethyl cellulose from oil palm trunk slabs: physicochemical properties and performance in acyclovir buccal films
Source: RSC Adv. 2026 Nov 10. Online ahead of print. doi: 10.1039/d6ra04937k (PMC13750149; doi:10.1039/d6ra04937k)
Supplement: Supplementary file 1 [file RA-OLF-D6RA04937K-s001.pdf]

Supplementary Information (SI) for RSC Advances.
This journal is © The Royal Society of Chemistry

Supplementary Information

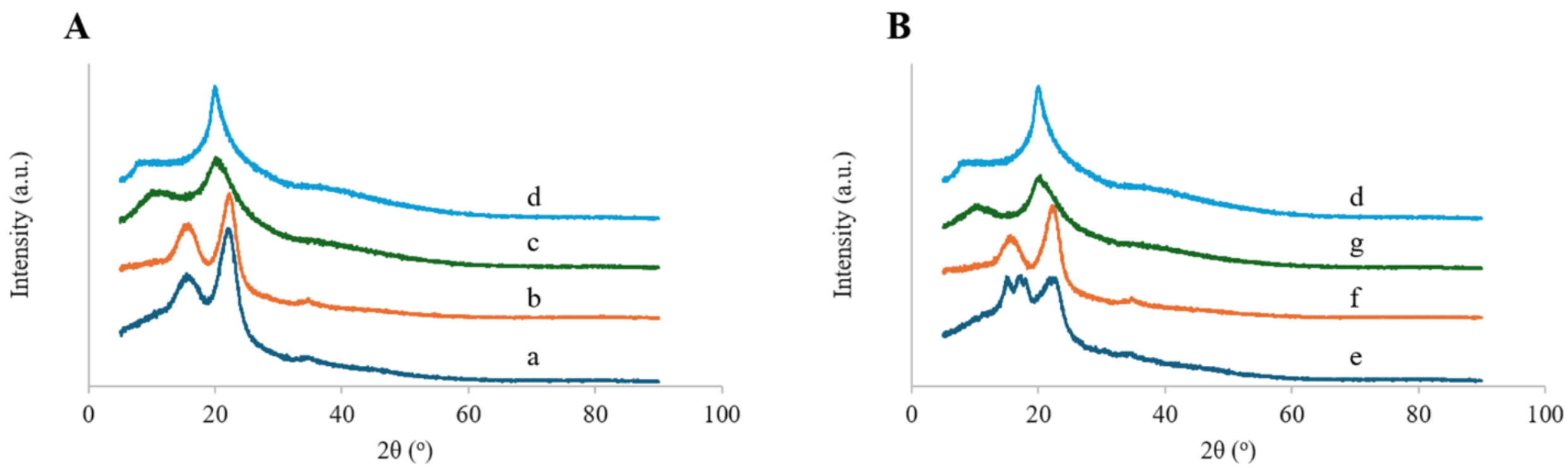

Material	Crystallinity (%)
**a: OPTS-b**	35.48
**b: Cellulose-b**	45.97
**c: CMC-b**	38.97
**d: CMC®**	30.17

Material	Crystallinity (%)
**e: OPTS-m**	23.01
**f: Cellulose-m**	44.05
**g: CMC-m**	37.21
**d: CMC®**	30.17

Fig. S1 XRD patterns of materials obtained during cellulose extraction and CMC synthesis from (A) OPTS-b and (B) OPTS-m, where a and e represent OPTS powder (OPTS-b and OPTS-m); b and f represent extracted cellulose (Cellulose-b and Cellulose-m); c and g represent synthesized CMC (CMC-b and CMC-m); and d represents commercial CMC (CMC®).

Table S1 Drug release kinetic parameters obtained from various release kinetic models (zero-order, first-order, Higuchi, and Korsmeyer–Peppas) for acyclovir release from buccal films prepared using different types of CMC: CMC-b (derived from the bottom section of OPTS), CMC-m (derived from the middle section of OPTS), and commercial CMC (CMC®).

Type of CMC	Zero-order		First-order		Higuchi		Korsmeyer-Peppas		
	R^2	k_0 (% h^{-1})	R^2	k_1 (h^{-1})	R^2	k_H (% $h^{-1/2}$)	R^2	k	*n*
CMC-b	0.935±0.098	19.580±2.408	0.938±0.052	0.456±0.134	0.955±0.039	50.399±4.264	0.960±0.045	27.136±5.920	0.902±0.130
CMC-m	0.948±0.049	20.774±1.007	0.975±0.015	0.485±0.057	0.976±0.005	54.727±0.823	0.984±0.020	34.422±3.023	0.955±0.190
CMC®	0.918±0.035	22.760±0.049	0.987±0.007	0.598±0.017	0.974±0.015	59.815±0.818	0.928±0.006	22.693±1.804	1.250±0.046
